# Application and Properties of Polyglycolic Acid as a Degradation Agent in MPU/HNBR Degradable Elastomer Composites for Dissolvable Frac Plugs

**DOI:** 10.3390/polym16020181

**Published:** 2024-01-08

**Authors:** Kai Cheng, Mingyang Yuan, Yupeng Zhang, Ningjing Sun, Bo Peng

**Affiliations:** 1 Beijing Key Laboratory for Greenhouse Gas Storage and CO2-EOR, Unconventional Petroleum Research Institute, China University of Petroleum (Beijing), Beijing 102249, China; 2020311105@student.cup.edu.cn (K.C.); 2022216612@student.cup.edu.cn (Y.Z.); 2023216611@student.cup.edu.cn (N.S.); 2 College of Engineering and Physical Sciences, Khalifa University of Science and Technology, Abu Dhabi 127788, United Arab Emirates; 2021211103@student.cup.edu.cn; 3 College of New Energy and Materials, China University of Petroleum (Beijing), Beijing 102249, China

**Keywords:** polyglycolic acid, MPU, HNBR, dissolvable elastomer, mechanical properties, dissolvable frac plugs

## Abstract

In this research, fully degradable elastomeric sealing materials were developed to enhance the environmental sustainability of oil and gas extraction. The modification of millable polyurethane rubber (MPU) with polyglycolic acid/hydrogenated nitrile butadiene rubber (PGA/HNBR) led to the synthesis of PGA@MPU/HNBR composite materials. The impact of varying monomer quantities on the mechanical properties, degradation behavior, degradation mechanisms, and thermal stability of these materials was investigated. Our findings illustrate that an increasing proportion of HNBR in the PGA@MPU/HNBR composite materials resulted in a decreased degradation rate. Simultaneously, higher HNBR content improved the thermal stability of the materials, while the inclusion of PGA reduced material hardness, rendering the composites more susceptible to swelling. At an HNBR content of 40 phr, MPU at 60 phr, and PGA at 6 phr, the composite material demonstrated the highest retention of mechanical properties at 31.3% following 168 h of hydrolysis at 100 °C. The degradation of the composite materials in 100 °C water primarily resulted from the hydrolysis of MPU’s ester groups, with HNBR remaining unaffected.

## 1. Introduction

In response to the escalating global demand for sustainable energy, the oil and gas industry is shifting focus from conventional to unconventional resources [1,2,3]. Unconventional resources, such as shale oil/gas, tightoil/gas, coalbed methane/oil, heavy oil, and oil sand, are plentiful and widespread distribution [4]. Advancements in technologies like horizontal drilling and multi-stage hydraulic fracturing have enabled commercial extraction from unconventional reservoirs [5,6,7]. Guided by the objective of carbon neutrality, the industry is prioritizing energy-efficient technologies [8]. The adoption of horizontal well multi-stage fracturing has become a key method in developing unconventional resources efficiently and sustainably. Compared to traditional vertical well fracturing, horizontal wells, with their greater deviation angles and more complex reservoir conditions, significantly enhance oil recovery and production [9]. Focusing on the technological aspect, fracturing plugs play an essential role in sustainable oil and gas development, helping reduce energy consumption and emissions [10,11,12]. And nowadays, the industry has seen a shift towards dissolvable frac plugs, which offer advantages like high strength, pressure resistance, and temperature tolerance while also being able to dissolve in appropriate fracturing fluids [13,14,15,16]. Particularly, dissolvable plugs made of degradable magnesium alloys have been a breakthrough in fracturing technology [17,18,19,20,21,22,23]. However, the development of the dissolvable rubber cylinder, a key component of these plugs, remains nascent, with many institutions keeping their research confidential. This has made research into degradable rubber sealing materials a focal point in oil production engineering. The exploration of novel degradable rubber materials is crucial for the advancement of this technology.

Downhole packers, integral to sealing mechanisms, necessitate the selection of appropriate soft materials of high molecular weight to ensure seal integrity, reduce operational disruptions, and mitigate costs and safety hazards in oil production. Materials such as millable polyurethane rubber (MPU) [13,24], hydrogenated nitrile butadiene rubber (HNBR) [25], nitrile butadiene rubber (NBR), fluoroelastomer (FKM), and ethylene propylene diene monomer (EPDM) are instrumental in these applications [26,27]. Notably, HNBR, with its extensive saturation and high-performance profile, is favored in oilfield isolation seals owing to its superior thermal stability, resistance to high temperatures, longevity, oil resilience, and defense against acid gas corrosion. Additionally, HNBR is prevalent in crafting seals for drilling pressure bridge plugs [25]. Despite its merits, HNBR’s insolubility in fracturing fluid poses retrieval challenges. Our previous investigations have identified MPU as a prime candidate for fabricating degradable rubber due to its composition of polyol soft segments and diisocyanate with chain extender hard segments [13]. Its elastomeric structure, featuring amide esters, urea, or ester groups, facilitates degradation through hydrolytic reactions, compromising the material’s mechanical integrity [28]. However, pure MPU sealants may dissolve too rapidly, precipitating seal failure [13]. Consequently, research into MPU modification is essential to tailor these materials for the fracturing sealant process.

The literature on degradable rubbers for petroleum applications has been limited in recent years. Commercially, such materials are categorized into two main types. The first employs specialized media to degrade high-performance rubber seals. These media are typically strong acids or bases, posing a risk of secondary environmental contamination. The second approach involves modifying a water-degradable rubber base to fulfill the requirements of the fracturing process, allowing the material to dissolve in fracturing fluid and be expelled through the wellbore along with the fluid. The dissolvable rubbers DR-043, DR-127, DR-129, DR-130, DR-131, and DR-132 were created by Yue et al. [16]. These six rubbers each have a variable rate of disintegration. According to the findings, the amount of time required for the coupon to shatter into little pieces is as follows: 16 days for DR-043, 4 days for DR-127, 6 days for DR-127, 2 days for DR-129, 24 h for DR-130, and 24 h for DR-131 and DR-132. In our previous work, we investigated the modification of MPU with HNBR, and the results showed that the HNBR/MPU composite material could maintain a high tensile strength in high-temperature media (100 °C) for 48–168 h to meet the sealing requirements of oil and gas exploitation [13]. However, the composite material hardened after immersion and could not dissolve into small, easily disposable pieces. In the previous works, a foundation has been established concerning the development of degradable rubber composites. Nevertheless, extant studies reveal a critical issue: the resultant rubber solutions often consist of rigid, large particulates that, upon flowback, precipitate wellbore obstructions [13,29,30,31]. To address this limitation, it is imperative to investigate the incorporation of degradative agents within the degradable rubber matrix to enhance its dissolution and prevent the formation of obstructive particulates.

Polyglycolic acid (PGA), a synthetic bio-based polymer, exhibits compelling biological characteristics, including biocompatibility and inherent biodegradability, positioning it as a polymer of interest within the biomedical field. Its application spectrum has been extensive, covering the development of absorbable sutures, tissue engineering scaffolds, and controlled drug delivery systems, underscoring its prominence in medical material research [32,33,34]. Moreover, the utilization of PGA exhibits promising potential in the field of packaging, particularly in the production of environmentally friendly biodegradable plastic bags [35,36,37]. PGA undergoes decomposition through the hydrolysis of ester linkages in its main chain, leading to the formation of smaller molecules. Eventually, PGA is further degraded into carbon dioxide via a biodegradation pathway, wherein the molecules are metabolized [38]. However, due to the high cost of PGA, it is seldom reported in the field of oil and gas field development. Therefore, based on the property that PGA can be completely degraded in water, it is possible to add an appropriate amount of PGA to the degradable rubber matrix so that the sealing material can achieve smaller dissolution and low hardness that can be easily returned to the wellbore along with the fracturing fluid.

In this work presented here, we aim to explore the development of degradable elastomer materials using a simple manufacturing process capable of normal operation during its working cycle, and the materials are degradable under specific conditions. To facilitate the close compatibility between MPU and HNBR and construct an interpenetrating network system, tetrahydrofuran was employed as a solvent to blend MPU and HNBR. Subsequently, PGA was incorporated as a degradation agent to enhance the dissolution of the composite materials in the MPU/HNBR blended. The mechanical properties of the PGA@HNBR/MPU composite materials were investigated, along with their degradation behavior in 100 °C water media. Finally, the dissolution behavior of composites with different ratios was characterized using infrared spectroscopy (FT-IR), thermogravimetric analysis (TG), scanning electron microscopy (SEM), and X-ray diffraction (XRD) to elucidate the underlying mechanism of PGAs role as a degrading agent in the rubber matrix.

## 2. Materials and Methods

### 2.1. Materials

Hydrogenated nitrile butadiene rubber (HNBR, Zetpol^®^ 1000 L) was sourced from Zeon Co., Ltd., Tokyo, Japan. Millable polyurethane rubber (MPU, UR^®^ 401) was supplied by Dongguan Huagongfosu New Material Co., Ltd., Dongguan, China. Analytical grade tetrahydrofuran (THF) and zinc oxide (ZnO) were procured from Sinopharm Chemical Reagent Co., Ltd., Shanghai, China. Polyglycolic acid (PGA, analytical grade, the weight-average molecular weight was 20 thousand g/mol) was acquired from Shandong Zengyi Bio-technology Co., Ltd., Jinan, China, while stearic acid (SA, chemical grade) was obtained from Tianjin Bodi Chemical Co., Ltd., Tianjin, China. Various grades of carbon black (N220, N550, N774, N990) were purchased from Shanghai Cabot Chemical Co., Ltd., Shanghai, China. The auxiliary vulcanizing agent triallyl isocyanurate (TAIC, chemical grade) and the vulcanizing agent dicumyl peroxide (DCP, chemical grade) were both secured from Shanghai Demo Chemical Co., Ltd., Shanghai, China, and Shanghai Fangruida Chemical Co., Ltd., Shanghai, China, respectively.

### 2.2. Preparation of PGA@MPU/HNBR Degradable Elastomer Composites

A series of blends comprising MPU and HNBR with different weight ratios (80/20, 70/30, and 60/40) were synthesized. The procedure entailed the dissolution of the MPU and HNBR blends in THF to generate a matrix material with a concentration of 100 parts per hundred rubber (phr). After the evaporation of the THF, the matrix was subsequently enriched with 5 phr of ZnO, 1 phr of SA, 60 phr of a carbon black mixture consisting of N550, N774, and N990, 6 phr of PGA as a degrading agent, 1 phr of TAIC as a co-vulcanizing agent, and 3 phr of DCP as a vulcanizing agent.

The experiment was conducted using a tiny Haake mixer, which was developed by the School of Mechanical and Electrical Engineering at Qingdao University of Science and Technology. The mixer was operated at a temperature of 70 °C, with a rotor speed of 35 rpm. The components were incorporated in a sequential manner, with the master batch being added first, followed by the addition of ZnO, SA, and the carbon black mixture. The compound underwent subsequent processing on an open rubber mill (XK-160, manufactured by Shanghai Shuangyi rubber & plastic machinery Co., Ltd., Shanghai, China) to facilitate further mixing, during which PGA, DCP, and TAIC were integrated. Following a 24 h period of resting at room temperature, the XLB-DQ type press vulcanizing machine, manufactured by Qingdao Yadong Machinery Group Co., Ltd., Qingdao, China, was employed for the initial stage of vulcanization. The vulcanization process was conducted under the following conditions: a temperature of 170 °C, a pressure of 15 MPa, and a duration of (*t*_90_ + 3) min. Subsequently, the vulcanized rubber underwent a second stage of vulcanization within an electric thermostatic drying oven, utilizing a temperature of 160 °C and a duration of 4 h.

### 2.3. Testing and Characterization

#### 2.3.1. Mechanical Properties

The mechanical properties of the samples were evaluated using an MZ-400D electrical universal tensile machine manufactured by Jiangsu Mingzhu Experimental Machinery Co., Ltd., Suzhou, China. Tensile strength measurements were carried out following the guidelines outlined in GB/T 528-2009. Dumbbell-shaped samples were used, and the tensile rate was set at 500 mm/min. The Shore A hardness was evaluated according to the standard GB/T 531-2008. The mechanical properties were evaluated under atmospheric conditions at room temperature.

#### 2.3.2. Dissolution Measurements

Dumbbell-shaped samples of PGA@MPU/HNBR degradable elastomer composites were prepared according to GB/T528-2009 and placed in a DK-98-II water bath (Tianjin Tester Co., Ltd., Tianjin, China) at 100 °C. Five samples were removed every 24 h for mechanical property testing as described in Section 2.3.1, with morphological changes recorded. The experiment was conducted for a duration of 168 h.

#### 2.3.3. Morphological Characterization

The morphology of the degradable elastomer PGA@MPU/HNBR was analyzed using scanning electron microscopy (SEM) (JSM-7610F, JEOL, Tokyo, Japan) at an accelerating voltage of 15 kV to examine the edge and interior regions of the samples. Prior to the examination, samples were rendered brittle in liquid nitrogen and subsequently sputter-coated with gold.

#### 2.3.4. Thermo-Gravimetric Analysis

The thermal stability of PGA@MPU/HNBR degradable elastomer composites was investigated using an SD-Q600 (TA Co., Ltd., New Castle, DE, USA) thermogravimetric analyzer under a nitrogen atmosphere. Approximately 10 mg of the sample was placed in a crucible and heated from 25 °C to 600 °C at a rate of 10 °C/min in the N_2_ atmosphere.

#### 2.3.5. The Fourier Transform Infrared Spectroscopy and X-ray Diffraction

Fourier transform infrared (FTIR) spectroscopy was conducted using a VERTEX-70 FTIR instrument (Bruker Corporation, Billerica, MA, USA) in the scanning range of 4000–500 cm^−1^, with 32 scans performed. X-ray diffraction (XRD) analysis was carried out using a D8 Advance X-ray Powder diffractometer (Bruker Corporation, Billerica, MA, USA), employing a scanning angle range of 3° to 50° and a scanning rate of 5 °/min.

## 3. Results and Discussion

### 3.1. Effect of PGA on the Mechanical Properties of MPU Materials

Our study primarily aims to examine the influence of different amounts of PGA on the mechanical characteristics of the MPU. We prepared PGA@MPU composite materials with PGA content of 3, 6, and 9 phr, respectively. Table 1 shows the effect of PGA content on the mechanical properties of MPU materials. It can be found that, from the experimental results, the initial tensile strength of the PGA@MPU composite material significantly dropped when compared with the blank MPU material without adding PGA. This was because the PGA with a low molecular weight was a brittle material, which was partially compatible with the soft segment of the MPU. After being subjected to tensile stress, the PGA had a gap with the rubber matrix, so the mechanical properties of the material could not be enhanced, and the macroscopic performance revealed a decline in the mechanical properties. With the increased PGA content, the tensile properties of the PGA@MPU composite material first increased and then decreased, and the elongation at break first decreased and then increased. In addition, we found that the mechanical properties of the three materials decreased after the addition of PGA, but since the prepared materials were used as a packer cylinder, we were more concerned about the tensile properties of the materials to meet the sealing requirements of high pressure. The tensile strength of the composite material added with 6 phr PGA was 22.06 MPa before being immersed in water, and the elongation at break was 239.5%, which was relatively close to the requirements of fracturing conditions.

After the PGA@MPU composite material was immersed in water at 100 °C for 24 h, it was found that the higher the PGA content, the lower the degradation rate of the composite material. The PGA@MPU composite material added with 3 phr PGA and immersed in water for 24 h could no longer be tested for material mechanical properties and was fragmented when an external force was applied. However, the PGA@MPU composite material added with 9 phr PGA still had a tensile strength of 7.42 MPa after being immersed in water for 24 h and subjected to the mechanical property test. This was because the excess PGA inhibited hydrolysis to a certain extent in the early stage. Part of the water reacted with the surface of the PGA first, and the remaining PGA and MPU entered the matrix of the material after blending. It took a long time for the explanation reaction to occur, so a part of the unhydrolyzed PGA played a physical reinforcement role in the PGA@MPU composite material. From the perspective of hydrolysis, the greater the content of the PGA, the better, but from the perspective of practical application, due to the general compatibility of PGA and MPU and the expensive PGA materials, in our study we finally selected the formula with the initial tensile strength of composite materials above 20 MPa, and the PGA content used in the next PGA@MPU/HNBR composite material was 6 phr.

### 3.2. Mechanical Properties of PGA@MPU/HNBR Composites Immersed in Water at 100 °C

**Figure 1:**
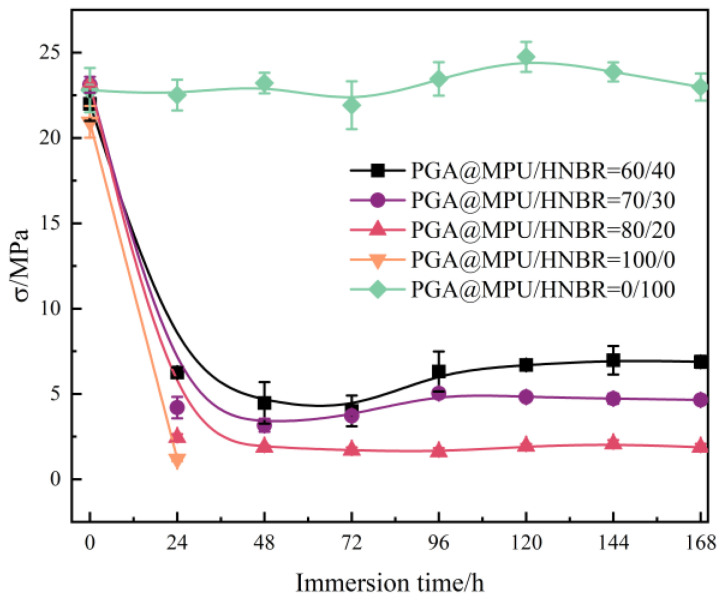
Effect of immersion time on the mechanical properties of PGA@HNBR/MPU composites (Note: PGA for all materials is 6 phr, the same as below).

After determining the content of the PGA, we prepared PGA@MPU/HNBR composite materials with different ratios of MPU/HNBR and tested the mechanical properties of the composite materials before and after being immersed in 100 °C water. Figure 1 shows the effect of immersion time on the mechanical properties of the PGA@MPU/HNBR composite materials.

The MPU had good degradation ability; its mechanical properties were lost after 24 h, and it dropped sharply from 20.94 MPa to 1.18 MPa. After being immersed for 48 h, the dumbbell-shaped sample was completely broken, so the mechanical property test could not be carried out. Thus, the composite material physically reinforced by pure MPU and pure carbon black could not meet the long-term fracturing sealing, and the property of the composite material modified by adding HNBR was greatly improved. However, there are significant limitations for water-degradable downhole applications for vulcanized rubber chemistries such as HNBR rubber materials. Because the backbone and monomer molecules of the HNBR are water-insoluble, the rubber may break down into water-insoluble chunks and remain in the wellbore. Since the soft segment of the MPU was hydrolyzed in hot water, the hydrophilic group moved to the outer surface of the material, so the colloid was sticky, while the mechanical properties of the HNBR hardly changed in hot water, and the tensile strength of the composite material after dissolution was mainly provided by the HNBR. Therefore, the larger the proportion of HNBR, the higher the tensile strength; the overall decline of different proportions of composite materials was like the rule of pure MPU composite materials at 0–24 h, and it tended to be stable after 24 h. After being immersed for 72 h, the mechanical property retention rates of the composite materials of three different ratios of MPU and HNBR (80/20, 70/30, 60/40) were only 7.3%, 16.1%, and 18.2%, respectively. After being immersed for 168 h, when the HNBR in the MPU/HNBR was increased from 20 phr to 40 phr, the tensile strength retention rate increased from 8.1% to 31.3%. When the amount of HNBR was 40 phr, the property of the PGA@MPU/HNBR composite material was the best, and it could still maintain a high tensile strength between 72 h and 168 h, and it still had a tensile strength of 6.88 MPa until 168 h. In comparison to our earlier works, while considering the identical content of HNBR-modified MPU, it exhibits a tensile strength of 9.8 MPa over a duration of 168 h [13]. The inclusion of the PGA has been observed to effectively decrease tensile strength. According to the literature [12,13,14,15], frac sealing materials need to maintain a high pressure during operation, up to 20–30 MPa, for particularly tough environments, so the initial tensile properties of the material need to meet the requirements of this condition. At the same time, after completing the fracture sealing and injecting the water medium, the sealing material needs to be degraded at a specific time, and the dissolved material is required to be small enough. Therefore, the addition of PGA can not only maintain a higher sealing pressure but also make the dissolved material smaller in shape. We will analyze the dissolution phenomenon of the material in detail in the next section.

### 3.3. Degradation Phenomenon of PGA@MPU/HNBR Composite Material

**Figure 2:**
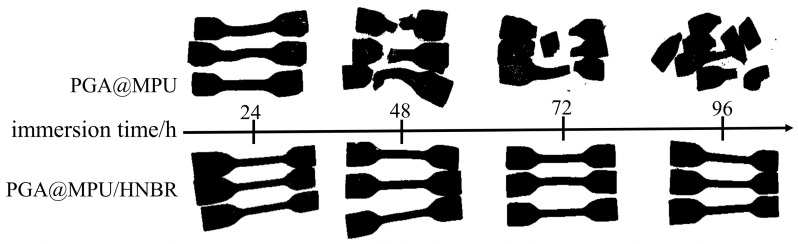
Influence of immersion time on the appearance and morphology of composite materials.

The comparison of the appearance of the PGA@MPU and PGA@MPU/HNBR composite material with immersion time is shown in Figure 2. Because the soft segment of the MPU contains ester groups, the molecular chain of the MPU undergoes an ester hydrolysis reaction at a high temperature, the mechanical properties are greatly reduced, and the hardness drops rapidly to below 50 so as not to maintain the shape of the dumbbell bar. After being immersed for 24 h, the dumbbell-shaped sample was deformed, the surface began to break, and filler particles could be seen inside. After being immersed for 48 h, the thinnest part of the dumbbell-shaped sample broke first and could no longer bear any external force. As the immersion time prolonged, the dumbbell-shaped sample changed from large fragments to small fragments, which could be broken into granules under a little external force. When HNBR was used to strengthen the MPU material, the MPU and HNBR formed an interpenetrating network. When the ester bond of the MPU was hydrolyzed, the HNBR did not cause the chemical bond to break. The PGA was finally dissolved and expanded the intermolecular gap by hydrophilic swelling, but it could not destroy the network structure of the HNBR. Therefore, the appearance of the dumbbell bar sample had a certain degree of swelling, but it still could not be broken by itself without an external force, and the PGA@MPU/HNBR composite material still maintained the original shape.

**Figure 3:**
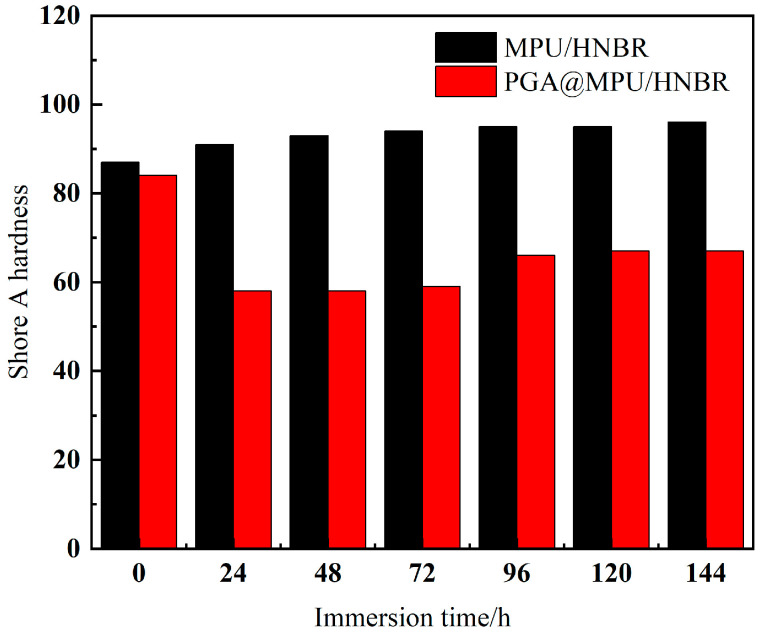
Influence of immersion time on the hardness of composite materials.

The change in hardness of the MPU/HNBR and PGA@MPU/HNBR composite material with immersion time is shown in Figure 3. With the increased immersion time, the overall hardness of the MPU/HNBR composite material was relatively stable and slowly rising, and there was no tendency to degrade. After the soft segment of the MPU was hydrolyzed, the HNBR skeleton and some hard segments of the MPU still supported the morphology. Under the high-temperature aging of 100 °C water, the composite material became aged and brittle, and its hardness continued to rise. In the later stage, the mechanical properties dropped until hands could break it, but the appearance and morphology still maintained the dumbbell-shaped shape. After the PGA@MPU/HNBR composite material was immersed in water, the Shore A hardness of the sample dropped precipitously from 84 to 58, and it proved that adding PGA can effectively reduce the hardness of the composite material after it was dissolved in water. After being immersed for 72 h, the hardness increased slowly and hardly changed, which was the same as the tensile law after being immersed in 100 °C water. It can be inferred that the effective effect of the PGA was about 72 h; after 72 h, the effect of the PGA was not obvious, the hardness did not continue to decrease, and the sample bar was more difficult to swell.

### 3.4. SEM Analysis of PGA@MPU/HNBR Composite Material before and after Degradation

**Figure 4:**
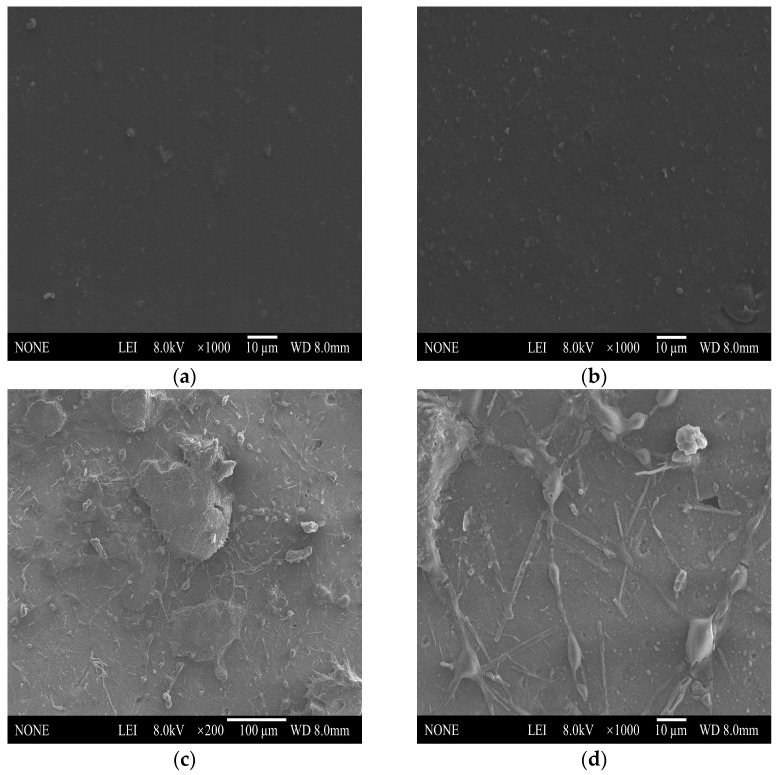
SEM images of the composite materials. (**a**) HNBR/MPU; (**b**) PGA@HNBR/MPU; (**c**,**d**) PGA@MPU/HNBR after hydrolysis.

On the one hand, we have analyzed the experimental photos of the PGA@MPU/HNBR composite material immersed in 100 °C water. On the other hand, to prove that the PGA@MPU/HNBR composite material was degraded microscopically, we used the SEM to observe the surface of the PGA@MPU/HNBR composite material before and after immersion, and the results are shown in Figure 4.

**Figure 5:**
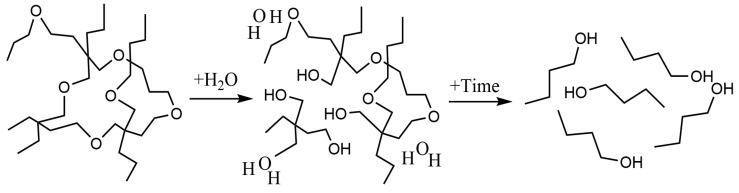
The hydrolysis reaction equation.

Figure 4a,b show the SEM images at the surface of the material samples. Figure 4a, not added with PGA, shows that after adding 6 phr PGA, due to the partial compatibility with the matrix material, uniform small particles appear on the surface of the matrix material, and it is worth mentioning that they are not observable to the naked eye. At the same time, as can be observed from Figure 4b, due to the small amount of PGA, it was evenly dispersed in the matrix without obvious agglomeration. Figure 4c,d shows the SEM images at the surface of the sample after immersion in high-temperature water at 100 °C, which shows that large particles, cracks, and holes appear on the surface of the composite material. This was because both the PGA and MPU contain ester groups, which could undergo a hydrolysis reaction under the catalysis of temperature. The hydrolysis reaction equation is shown in Figure 5. Observing the higher resolution of Figure 4d, we found that the hydrolysis reaction of the PGA and MPU occurs under the catalysis of temperature. A part of the PGA absorbs water and expands, and the PGA changes from small particles to large particles. Another part of the PGA has been hydrolyzed, generating small molecules containing hydroxyl or carboxyl groups, which enter into the solution water and leave pores on the surface of the material. After the hydrolysis of the MPU, many cracks appeared on the surface of the matrix material, but because HNBR still supports the skeleton of the material, the SEM observation of the material still maintains the structural integrity. Therefore, the SEM does not observe the kind of manifestation of material fragmentation that occurs with macroscopic degradation phenomena.

### 3.5. FT-IR Analysis of PGA@MPU/HNBR Composite Materials

**Figure 6:**
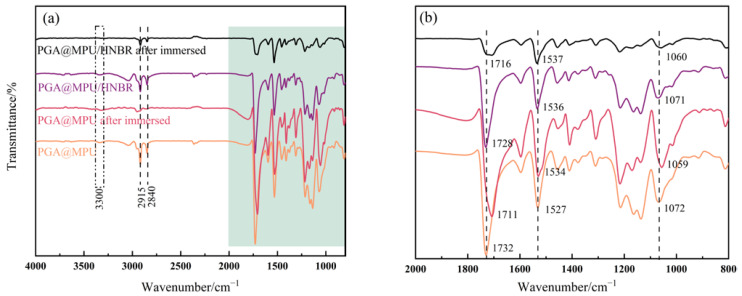
FT-IR spectra. (**a**) Infrared spectra of the PGA@MPU/HNBR composite materials before and after immersion; (**b**) partial enlargement.

The FT-IR of the PGA@MPU composite material and PGA@MPU/HNBR composite material before and after degradation in water at 100 °C is shown in Figure 6. The absorption peaks at 2915 cm^−1^ and 2840 cm^−1^ in the figure represent stretching vibration peaks of -CH_3_, -CH_2_, and -CH. The absorption peak at 1732 cm^−1^ was the stretching vibration of C=O in the ester group, and 1527 cm^−1^ was the -NH bending vibration peak in the isocyanate group in the MPU [13]. Before degradation, the absorption peak of the urethane group of the composite material added with HNBR was weakened. At the same time, the HNBR enhanced the hydrogen bonding of the composite material, the decrease in electron cloud density strengthened the C=O chemical bond, and the wave number decreased from 1732 cm^−1^ to 1728 cm^−1^. After immersion and degradation, part of the urethane underwent a hydrolysis reaction. The position of the C=O vibration was blue-shifted, the PGA@MPU composite material was reduced from 1728 cm^−1^ to 1716 cm^−1^, and the PGA@MPU/HNBR composite material was changed from 1732 cm^−1^ to 1711 cm^−1^. In addition, due to the drying of the material during the FT-IR testing, we found only very weak hydroxyl absorption peaks at the 3300 cm^−1^ attachment in the spectrum.

### 3.6. Thermal Stability of PGA@MPU/HNBR Composite Material

**Figure 7:**
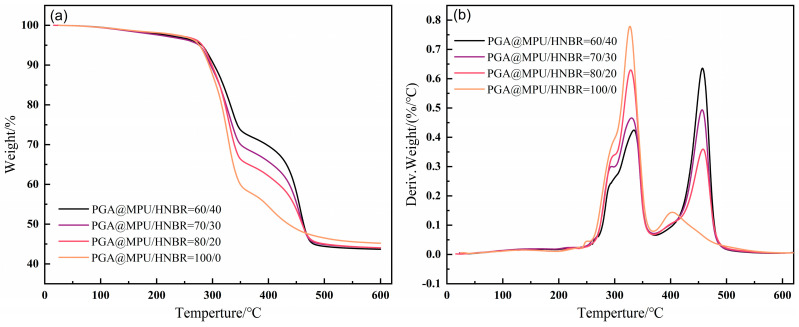
Thermogravimetric curves of the PGA@MPU/HNBR composite materials. (**a**) TG, (**b**) DTG.

Figure 7 shows the TG curves of different proportions of the PGA@MPU/HNBR composite material before immersion in water at 100 °C. The PGA@MPU composite material only had one weightless step, while the PGA@MPU/HNBR composite material had two weightless steps. This was because the structure of the rubber was destroyed by thermal cracking during the temperature rise process, and the structure of the MPU lost weight rapidly at 300~350 °C, while the structure of the HNBR lost weight rapidly at 420~480 °C. The heat resistance of the HNBR was better than that of the MPU. As the HNBR content increased, the larger the proportion in the composite material, the slower the thermal degradation of the composite material.

**Figure 8:**
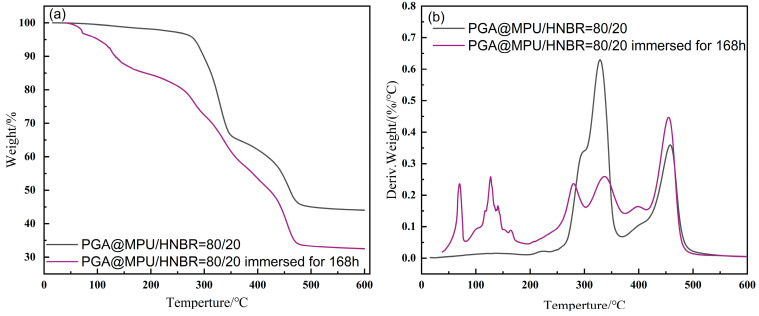
TG curve of the PGA@HNBR/MPU composite materials before and after immersion. (**a**) TG, (**b**) DTG.

The TG curve of the immersed PGA@MPU/HNBR composite material is shown in Figure 8. The immersed composite material had no obvious large steps but had many small staged weight losses. This was because the degraded composite material was fragmented into many hard-segment crystals with different molecular weights. As the temperature rose, the quality of the immersed composite material plummeted, and there was no high-temperature-resistant structural support. However, the composite material before immersion hardly lost weight before 280 °C, which was due to the good thermal stability of the interpenetrating network structure of the MPU and HNBR; it can support high temperatures over a short period of time. After the composite material was immersed in high-temperature medium water, the ester group was hydrolyzed, including the ester group in the urethane, so the stability of the material was decreased. It proved that its structure had almost no urethane bond. After being immersed, the soft segment of the composite material dissolved in water, the soft segment structure of MPU was destroyed, the material became very brittle and inelastic, they were all supported by HNBR and the hard segment of the MPU, and the whole material was broken when external force was applied. In addition, as observed in Figure 8, the weight loss of the PGA@HNBR/MPU composite materials after 168 h of immersion differs by about 10% compared to the one not immersed. This observation confirms that approximately 10% of the soft segments in the composite materials are lost due to immersion in water.

### 3.7. XRD Test of PGA@MPU/HNBR Composite Material

**Figure 9:**
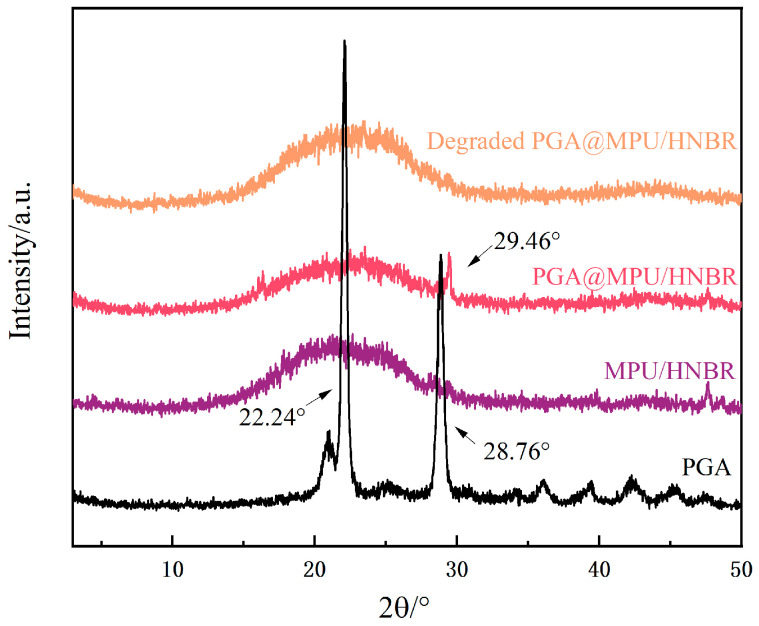
XRD patterns of the PGA, HNBR/MPU, PGA@HNBR/MPU, and PGA@HNBR/MPU after hydrolysis.

To assess the structural changes in the PGA within the PGA@MPU/HNBR composite material, particularly its behavior during the hydrolysis process, we conducted X-ray diffraction (XRD) analysis. The results are presented in Figure 9. While MPU/HNBR is inherently amorphous and does not exhibit distinct diffraction patterns, PGA, with its crystalline structure, provided specific peaks that we could monitor for changes. The crystal diffraction peaks of PGA at the (110) plane and (020) plane appeared around 2θ of 22.24° and 28.76°, respectively [36]. Upon adding PGA to the amorphous matrix of the MPU/HNBR, the composite largely retained its amorphous character, evidenced by the absence of significant diffraction peaks. A minor peak at 29.46° was observed, suggesting the presence of PGA. Notably, this peak shifted compared to pure PGA, possibly due to the interaction with other components in the composite.

After hydrolysis, the XRD pattern of the PGA@MPU/HNBR composite closely resembled that of the original MPU/HNBR, indicating the hydrolysis of PGA. The original minor peak associated with PGA disappeared, suggesting changes in the crystalline structure of the PGA or its complete degradation, rendering it non-crystalline. This shift supports the conclusion that the PGA underwent significant structural changes during the hydrolysis process, which were sufficiently impactful to alter its crystalline arrangement.

## 4. Conclusions

In the current study, we investigated the degradation behavior of a novel multicomponent blend system of PGA, HNBR, and MPU formulated to decelerate the degradation rate of MPU. Our comprehensive analysis, including FTIR, XRD, SEM, and TGA, confirmed that hydrolysis occurred within this composite material under high-temperature aqueous conditions at 100 °C. The salient conclusions from our study are as follows:

(1) The addition of PGA to MPU leads to a decline in the initial mechanical properties of the PGA@MPU composites. The optimal PGA content for balancing the degradation rate and mechanical properties was determined to be 6 phr.
(2) PGA contributed to enhanced hydrolytic degradation and a reduction in material hardness. Hydrolysis primarily affected the ester groups in both PGA and MPU, with complete degradation of MPU ester groups within 24 h and PGA ester groups degrading over approximately 72 h.
(3) The PGA@MPU/HNBR composites exhibited a marked decrease in mechanical properties within the first 24 h of immersion, stabilizing after that—a higher HNBR content correlated with a better retention of mechanical properties.
(4) Following initial degradation, the PGA@MPU/HNBR composites underwent late-stage colloidal hardening, maintaining sufficient strength for potential use in dissolvable frac plugs designed for controlled fragmentation.

These findings advance our understanding of degradation behaviors in such multicomponent blend systems and offer a strategic approach to engineering materials with controlled degradability for hydraulic fracturing applications.

## Figures and Tables

**Table 1:** Mechanical properties of PGA@MPU composites.

PGA/phr	0	3	6	9
σ/MPa	27.3 ± 0.82	18.3 ± 0.92	22.1 ± 0.99	19.8 ± 1.4
ε/%	220 ± 6.6	279 ± 10.8	240 ± 13.95	276 ± 19.32
H/Shore A	89	78	81	81
σ^2^/MPa	/	/	1.18 ± 0.04	7.42 ± 0.45

Note: σ, tensile strength; ε, elongation at break; H, hardness; σ^2^, tensile strength after 24 h immersion.

## Data Availability

The data that support the findings of this study are available from the corresponding author upon reasonable request.

## References

[1] McGlade C., Speirs J., Sorrell S. (2013). Unconventional Gas—A Review of Regional and Global Resource Estimates. Energy.

[2] Davies R.J., Almond S., Ward R.S., Jackson R.B., Adams C., Worrall F., Herringshaw L.G., Gluyas J.G., Whitehead M.A. (2014). Oil and Gas Wells and Their Integrity: Implications for Shale and Unconventional Resource Exploitation. Mar. Pet. Geol..

[3] Jia C. (2017). Breakthrough and Significance of Unconventional Oil and Gas to Classical Petroleum Geology Theory. Pet. Explor. Dev..

[4] Zou C., Qiu Z., Zhang J., Li Z., Wei H., Liu B., Zhao J., Yang T., Zhu S., Tao H. (2022). Unconventional Petroleum Sedimentology: A Key to Understanding Unconventional Hydrocarbon Accumulation. Engineering.

[5] Wenbin C., Zhaomin L., Xialin Z., Bo Z., Qi Z. (2009). Horizontal Well Fracturing Technology for Reservoirs with Low Permeability. Pet. Explor. Dev..

[6] Li G., Sheng M., Tian S., Huang Z., Li Y., Yuan X. (2012). Multistage Hydraulic Jet Acid Fracturing Technique for Horizontal Wells. Pet. Explor. Dev..

[7] Baki S., Alzamil A., Habiballah Z., Shariff E., Alowaid A., Alnakhli W., Alshammari H., Mahfouz M. Frac Plug Evaluation to Improve Operations Efficiency. Proceedings of the SPE Middle East Oil, Gas and Geosciences Show.

[8] Wang Z., Li S., Jin Z., Li Z., Liu Q., Zhang K. (2023). Oil and Gas Pathway to Net-Zero: Review and Outlook. Energy Strategy Rev..

[9] Cao X., Wang M., Kang J., Wang S., Liang Y. (2020). Fracturing Technologies of Deep Shale Gas Horizontal Wells in the Weirong Block, Southern Sichuan Basin. Nat. Gas Ind. B.

[10] Zhao C., Xu Z. (2023). CFD Simulation of Flow Interference between Multi-Stage Fractures along a Horizontal Wellbore. Geoenergy Sci. Eng..

[11] Trabelsi H., Seibi A., Liu N., Boukadi F., Trabelsi R. (2021). Bridge Plug Drillouts Cleaning Practices—An Overview. Nat. Resour..

[12] Chen L., Wei R., Wei S., Wang X. (2019). Application of Bionic Technologies on the Fracturing Plug. Biomimetics.

[13] Cheng K., Shang L., Li H., Peng B., Li Z. (2023). A Novel Degradable Sealing Material for the Preparation of Dissolvable Packer Rubber Barrel. J. Macromol. Sci. Part A.

[14] Zhao J., Ren L., Jiang T., Hu D., Wu L., Wu J., Yin C., Li Y., Hu Y., Lin R. (2022). Ten Years of Gas Shale Fracturing in China: Review and Prospect. Nat. Gas Ind. B.

[15] Fripp M., Walton Z. Fully Dissolvable Frac Plug Using Dissolvable Elastomeric Elements. Proceedings of the SPE Middle East Oil & Gas Show and Conference.

[16] Yue W., Ren J., Yue J., Cheng P., Dunne T., Zhao L., Patsy M., Nettles D., Liu Y., Liu H. High Temperature Dissolvable Materials Development for High Temperature Dissolvable Plug Applications. Proceedings of the SPE Annual Technical Conference and Exhibition.

[17] Geng Z., Xiao D., Chen L. (2016). Microstructure, Mechanical Properties, and Corrosion Behavior of Degradable Mg-Al-Cu-Zn-Gd Alloys. J. Alloys Compd..

[18] Liu L., Yu S., Niu Y., Liu E. (2020). Preparation and Properties of Hollow Glass Microspheres Reinforced Mg Alloy Degradable Composites. J. Alloys Compd..

[19] Liu B., Yang Y., Zhang Y., Du H., Hou L., Wei Y. (2020). Investigation of Rapidly Decomposable AZ91–RE–xCu (X = 0, 1, 2, 3, 4) Alloys for Petroleum Fracturing Balls. J. Phys. Chem. Solids.

[20] Niu H., Deng K., Nie K., Wang C., Liang W., Wu Y. (2020). Degradation Behavior of Mg-_4_Zn-_2_Ni Alloy with High Strength and High Degradation Rate. Mater. Chem. Phys..

[21] Sun J. (2022). A Review on Magnesium Alloys for Application of Degradable Fracturing Tools. J. Magnes. Alloys.

[22] Liu Y.H., Zhang Z.R., Wang J., Li Y., Li H.X., Jia L.Y., Wang J.H., Zhang J.S. (2023). A Novel Mg-Gd-Y-Zn-Cu-Ni Alloy with Excellent Combination of Strength and Dissolution via Peak-Aging Treatment. J. Magnes. Alloys.

[23] Liu Y., Zhang Z., Zhang J., Li Y., Jia L., Liu B., Li H. (2023). A Dissolvable Mg-Gd-Y-Zn-Cu Alloy Possessing the Highest Yield Strength for the Fabrication of Fracturing Plugging Tools. Mater. Lett..

[24] Duran M.M., Moro G., Zhang Y., Islam A. (2023). 3D Printing of Silicone and Polyurethane Elastomers for Medical Device Application: A Review. Adv. Ind. Manuf. Eng..

[25] Hassani F., Faisal N.H., Nish R., Rothnie S., Njuguna J. (2022). The Impact of Thermal Ageing on Sealing Performance of HNBR Packing Elements in Downhole Installations in Oilfield Wellhead Applications. J. Pet. Sci. Eng..

[26] Jiang X., Yuan X., Guo X., Zeng F., Wang H., Liu G. (2023). Study on the Application of Flory–Huggins Interaction Parameters in Swelling Behavior and Crosslink Density of HNBR/EPDM Blend. Fluid Phase Equilibria.

[27] Guo J., Xu P., Lv J., Han X., Sun Y., Hou D., Yuan Z., Li C. (2023). Ageing Behaviour and Molecular/Network Structure Evolution of EPDM/Carbon Black Composites under Compression and in Thermal-Oxidative Environments. Polym. Degrad. Stab..

[28] Ramirez M., Miller K.R., Soucek M.D. (2014). Linking of Oligoesters Hydrolysis to Polyurethane Coatings. J. Appl. Polym. Sci..

[29] Walton Z., Fripp M., Merron M. Dissolvable Metal vs. Dissolvable Plastic in Downhole Hydraulic Fracturing Applications. Proceedings of the Offshore Technology Conference.

[30] Burdzy M.P. Aqueous Degradable Polyurethane Elastomers for Oil & Gas Applications. Proceedings of the Offshore Technology Conference.

[31] Ren J., Cheng P., Wang X. Dissolvable Rubbers Development and Its Applications in Downhole Tools. Proceedings of the SPE Middle East Oil & Gas Show and Conference.

[32] Mhiri S., Mignard N., Abid M., Prochazka F., Majeste J.-C., Taha M. (2017). Thermally Reversible and Biodegradable Polyglycolic-Acid-Based Networks. Eur. Polym. J..

[33] Feng P., Shen S., Shuai Y., Peng S., Shuai C., Chen S. (2023). PLLA Grafting Draws GO from PGA Phase to the Interface in PLLA/PGA Bone Scaffold Owing Enhanced Interfacial Interaction. Sustain. Mater. Technol..

[34] Shibutani M., Nagahara H., Fukuoka T., Iseki Y., Okazaki Y., Hirakawa K., Ohira M. (2021). Prevention of Anastomotic Leakage Using a Polyglycolic Acid Sheet in Double-Stapling Technique Anastomosis for Rectal Surgery. Ann. Med. Surg..

[35] Xu P., Tan S., Niu D., Wang Q., Liu T., Yang W., Ma P. (2023). Effect of Temperatures on Stress-Induced Structural Evolution and Mechanical Behaviors of Polyglycolic Acid/Polycaprolactone Blends. Polymer.

[36] Xu Z., Dong Y., Yang Y., Zhu J. (2023). Mechanical, Barrier, and Biodegradable Properties of Poly(Butylene Adipate-*Co*-Terephthalate)/Polyglycolic Acid Blends Prepared by Reactive Extrusion. ACS Appl. Eng. Mater..

[37] Kwon H.-J., Jang J., Koh W.-G., Lee J.-Y., Hwang K. (2023). Ductile Effect of PGA/PCL Blending Plastics Using a Novel Ionic Chain Extender with Non-Covalent Bonds. Polymers.

[38] Li C., Meng X., Gong W., Chen S., Xin Z. (2023). Kinetic, Products Distribution, and Mechanism Analysis for the Pyrolysis of Polyglycolic Acid toward Carbon Cycle. Fuel.

